# Classification Model for Epileptic Seizure Using Simple Postictal Laboratory Indices

**DOI:** 10.3390/jcm12124031

**Published:** 2023-06-13

**Authors:** Sun Jin Jin, Taesic Lee, Hyun Eui Moon, Eun Seok Park, Sue Hyun Lee, Young Il Roh, Dong Min Seo, Won-Joo Kim, Heewon Hwang

**Affiliations:** 1 Department of Neurology, Wonju Severance Christian Hospital, Yonsei University Wonju College of Medicine, Wonju 26426, Republic of Korea; h2429609@naver.com (S.J.J.); pes3954@naver.com (E.S.P.); arcticmoon17@naver.com (S.H.L.); 2 Division of Data Mining and Computational Biology, Institute of Global Health Care and Development, Wonju 26426, Republic of Korea; ddasic123@yonsei.ac.kr; 3 Department of Family Medicine, Yonsei University Wonju College of Medicine, Wonju 26426, Republic of Korea; hyuneui@yonsei.ac.kr; 4 Department of Emergency Medicine, Yonsei University Wonju College of Medicine, Wonju 26426, Republic of Korea; md.youngilroh@yonsei.ac.kr; 5 Department of Medical Information, Yonsei University Wonju College of Medicine, Wonju 26426, Republic of Korea; dmseo@yonsei.ac.kr; 6 Department of Neurology, Gangnam Severance Christian Hospital, Yonsei University College of Medicine, Seoul 06273, Republic of Korea; kzoo@yuhs.ac

**Keywords:** seizure, syncope, serum, ammonia, bayes approach

## Abstract

Distinguishing syncope from epileptic seizures in patients with sudden loss of consciousness is important. Various blood tests have been used to indicate epileptic seizures in patients with impaired consciousness. This retrospective study aimed to predict the diagnosis of epilepsy in patients with transient loss of consciousness using the initial blood test results. A seizure classification model was constructed using logistic regression, and predictors were selected from a cohort of 260 patients using domain knowledge and statistical methods. The study defined the diagnosis of seizures and syncope based on the consistency of the diagnosis made by an emergency medicine specialist at the first visit to the emergency room and the diagnosis made by an epileptologist or cardiologist at the first outpatient visit using the International Classification of Diseases 10th revision (ICD-10) code. Univariate analysis showed higher levels of white blood cells, red blood cells, hemoglobin, hematocrit, delta neutrophil index, creatinine kinase, and ammonia levels in the seizure group. The ammonia level had the highest correlation with the diagnosis of epileptic seizures in the prediction model. Therefore, it is recommended to be included in the first examination at the emergency room.

## 1. Introduction

Transient loss of consciousness is a common reason for emergency department visits, and epileptic seizures and syncope are the two primary causes of sudden loss of consciousness. The treatment of patients who visit the emergency room due to sudden loss of consciousness begins with classification into syncope and seizure. Discrimination is made by listening to a detailed history, completing a questionnaire, and identifying the patients’ characteristics [1]. It is not difficult for neurologists to differentiate between the two when identifying typical clinical symptoms. However, patients who visit the emergency room with decreased consciousness cannot express their symptoms properly, and it is difficult to make an accurate evaluation solely based on the information gathered from listening to the symptoms reported by eyewitnesses.

Although the clinical manifestations of seizures and syncope can be similar, their causes are different; therefore, establishing an appropriate treatment direction at the early stage of admission to the emergency room is important for prognosis. Syncope is the most common symptom mistaken for epileptic seizure [2]. Jerky movements, post-ictal confusion, and tongue biting are rare but can also occur in syncope, confused with seizure [3]. Therefore, it is important to use objective indicators to distinguish between syncope and seizures. By incorporating objective physiological measures, we can establish a more reliable classification of patients who present with similar symptoms.

There have been studies conducted to predict epileptic seizures in the initial classification of patients with decreased consciousness who visit the emergency room using blood tests [4,5]. Creatine phosphokinase (CK) has been known to be helpful in diagnosis when differentiating between epileptic seizures and psychogenic non-epileptic seizures (PNES) [6]. It is especially helpful in differentiating generalized tonic-clonic seizures (GTCS) when elevated. Contrarily, in a Korean study, the serum level of brain-type natriuretic peptide (BNP) did not show usefulness in differentiating seizure and vasovagal syncope in the clinical setting [7,8].

Studies have shown that a temporary increase in plasma ammonia levels in patients with loss of consciousness can help distinguish between seizures and syncope in the emergency room. However, there is still insufficient research [9]. Serum lactate dehydrogenase (LDH), an enzyme that catalyzes the conversion of lactate and pyruvate, is known to be elevated in patients with acute illness accompanied by seizures. There are also studies suggesting that neuron-specific enolase (NSE) elevation can be helpful in distinguishing between seizures and syncope [10]. Furthermore, white blood cell (WBC) counts can be temporarily elevated during seizures, especially GTCS [11,12,13]. Moreover, a previous study showed that changes in white blood cell count result in abnormal electroencephalogram (EEG) findings [14].

Reducing unnecessary evaluation and treatment is possible if we can predict whether the cause of transient loss of consciousness is an epileptic seizure using basic blood test results. Several studies have been conducted to identify individual predictive factors associated with transient loss of consciousness conditions including seizure and syncope. However, research on the combinatory effects of seizure-related laboratory features is still lacking. The purpose of this study is to differentiate epileptic seizure by analyzing the initial basic blood test results of patients who visited the emergency room because of transient loss of consciousness.

## 2. Materials and Methods

### 2.1. Participants

The study retrospectively analyzes patients experiencing transient loss of consciousness who visited the outpatient clinic more than once after leaving the emergency room of Wonju Severance Christian Hospital (WSCH) between 1 January 2019 and 30 June 2022. The diagnosis of seizures and syncope was confirmed if the initial diagnosis made by the emergency medicine specialist during the first emergency room visit was consistent with the diagnosis made by the epileptologist or cardiologist during the first outpatient visit, based on the International Classification of Diseases 10th revision (ICD-10) code. The codes used for diagnosis were R55 for syncope and G40, G41, and G56 for seizures.

**Figure 1:**
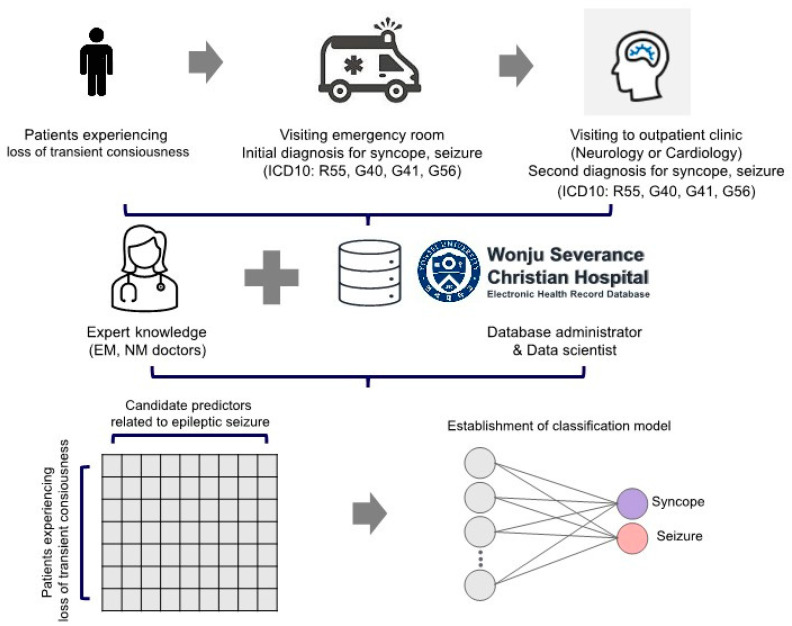
Flow Chart of Study Population Selection Process; EM = Emergency Medicine; NM = Neurology.

The initial dataset was built using an automatic platform, similar to previous studies, by incorporating patients’ hospital visit dates and diagnoses [15,16,17]. First, we identified 487 patients aged 18 years or older. Only those with complete clinical and laboratory data were enrolled in the study, resulting in 262 patients with transient loss of consciousness. Finally, we selected 260 patients whose diagnoses of either seizure or syncope were consistent between the epileptologist or cardiologist at the first outpatient visit, based on ICD-10 codes and the emergency medicine specialist. The presence of medical comorbidities, including cerebrovascular disease (ischemic or hemorrhagic), neurodegenerative disease (dementia, Parkinson’s disease), hypertension, diabetes mellitus, dyslipidemia, cardiovascular disease, cardiac arrhythmia, liver disease, renal disease, and thyroid disease, was manually supplemented based on the medical records by medical doctors. Blood tests performed within 8 h after visiting the emergency room were added to the dataset using an automatic platform (Figure 1).

### 2.2. Determination of Outcome and Predictor Variables Related to Epileptic Seizure

**Figure 2:**
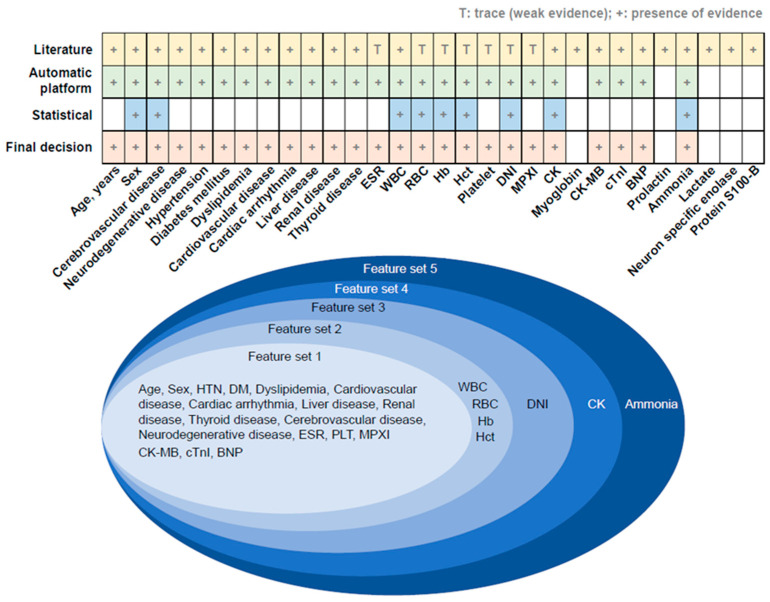
Predictor and feature set selection based on literature review and statistical methods; ESR = erythrocyte sedimentation rate; WBC = white blood cell; RBC = red blood cell; Hb = hemoglobin; Hct = hematocrit; DNI = delta neutrophil index; MPXI = myeloperoxidase index; CK = creatinine kinase; CK-MB = creatinine kinase-myoglobin binding; cTnI = cardiac troponin I; BNP = brain-type natriuretic peptide; HTN = hypertension; DM = diabetes mellitus.

Two epileptologists selected the initial predictors of seizures or syncope based on a literature review. A dataset was built using an automatic platform to include as many variables as possible, primarily selected through a literature review of the hospital database. Next, we selected the variables that most participants possessed. After removing the sparse predictive factors, a dataset was constructed to build the classification model (Figure 2).

The predictors were classified into continuous and non-continuous variables, and variables were selected using statistical techniques such as the *t*-test or chi-square test. Variables that were not statistically significant but had a tendency and were reported in many studies to be associated with seizures were finally added through discussions with an expert panel.

### 2.3. Cross-Validation to Compare Predictive Performance

Cross-validation (CV) of 200 × 5-fold was implemented to compare the predictive performance of the four feature sets for the epileptic seizure. The following steps were conducted. Step (1): All datasets were equally divided into five subsets. Step (2): Four sub-datasets were arranged as the training dataset, and a remnant sub-dataset was categorized as the testing dataset. Step (3): Using the training dataset and logistic regression (LR), a classification model was established. Step (4): Based on the testing dataset, predictive accuracy was measured. Step (5): Steps 3 to 4 were iterated five times (5-fold CV). Step (6): Steps 1 to 5 were repeated 200 times (200 × 5-fold CV).

### 2.4. Statistical Analysis

The clinical characteristics of continuous and categorical variables according to the two types of transient loss of consciousness were compared using Student’s *t*-test and chi-square test, respectively. Prediction performance was measured based on specificity, sensitivity, and their combinatory index, referred to as the Receiver Operating Characteristic (ROC) curve and area under the ROC curve. All statistical analyses and the establishment of the prediction model were performed using the R language. Statistical significance was set at *p* < 0.05.

## 3. Results

In this study, 260 patients with transient loss of consciousness were included, with 170 and 90 patients categorized into the syncope and seizure groups, respectively.

The mean age of the syncope group was 59.5 ± 1.54 years, and the mean age of the seizure group was 55.4 ± 1.76 years, but the difference was not statistically significant (*p* = 0.105). Male patients comprised 46.5% of the syncope group and 60% of the seizure group, with a trend toward significance (*p* = 0.052). The prevalence of cerebrovascular disease (CVD) was significantly higher in the seizure group (34.4%) than in the syncope group (13.5%) (*p* < 0.001). There were no significant differences between the two groups regarding neurodegenerative diseases, hypertension, diabetes mellitus, dyslipidemia, cardiovascular diseases, cardiac arrhythmia, liver diseases, renal diseases, or thyroid diseases.

Several statistically significant findings emerged from the analysis of hematological and biochemical markers. The seizure group showed a significantly higher white blood cell count (9.9 ± 0.48 × 10^3^/μL) compared to the syncope group (7.6 ± 0.23 × 10^3^/μL) (*p* < 0.001). Red blood cell (RBC) count was also significantly higher in the seizure group (4.5 ± 0.07 × 10^6^/μL) than in the syncope group (4.4 ± 0.04 × 10^6^/μL) (*p* = 0.029). Additionally, the seizure group had slightly elevated hemoglobin levels (14 ± 0.19 g/dL vs. 13.4 ± 0.15 g/dL) and a higher hematocrit level (42.4 ± 0.62% vs. 40 ± 0.4%) compared to the syncope group (*p* = 0.013 and *p* = 0.002, respectively).

The seizure group exhibited a significantly higher delta neutrophil index (DNI) (0.7 ± 0.18%) compared to the syncope group (0.3 ± 0.06%) (*p* = 0.017). However, no significant differences were found in erythrocyte sedimentation rate (ESR), platelet counts, and mean platelet volume index (MPXI) between the two groups (*p* > 0.05).

Notably, the seizure group exhibited a significantly higher ammonia level (93.9 ± 12.79 µg/dL) compared to the syncope group (29.2 ± 1.45 µg/dL) (*p* < 0.001). Moreover, CK levels were significantly elevated in the seizure group (336.1 ± 79.45 IU/L) compared to the syncope group (132 ± 12.75 IU/L) (*p* = 0.013). However, no significant differences were observed in creatine kinase-myoglobin binding (CK-MB), cardiac troponin I, and BNP levels between the two groups (*p* > 0.05) (Table 1).

**Figure 3:**
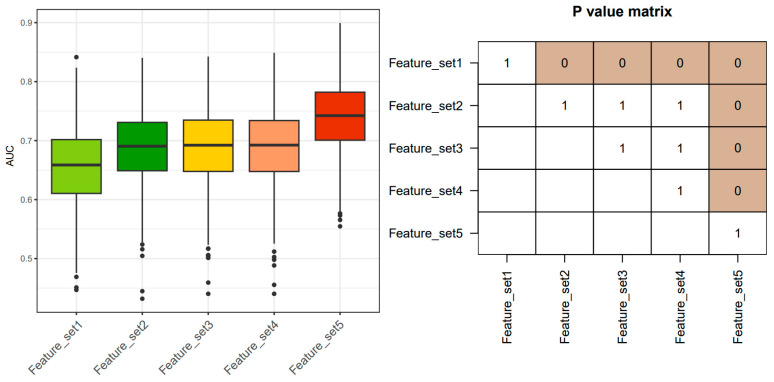
Comparison of performance of epileptic seizure prediction models based on five feature sets.

Initially, a literature-based review was conducted to identify 30 potential predictors related to epileptic seizures, as listed in Table S1. However, certain serum biomarkers, including myoglobin, prolactin, lactate, NSE, and protein S100-B, which are not routinely examined in clinical practice and had a high number of missing values, were excluded from the automatic platform, as depicted in Figure 2. Subsequently, statistical methods such as *t*-tests and chi-square tests were utilized to evaluate 25 variables as potential predictors of epileptic seizures. Finally, 25 candidate predictors were identified by consolidating expert knowledge and utilizing information from the WSCH database. Considering the statistical results, we arranged them into five feature combinations and conducted a comparative analysis of the predictive performance among the five sets (Figure 3).

We generated 1000 Area Under the Curve (AUC) values for each of the five lists of seizure-related predictors after conducting 250 × 4 CV on the real hospital electronic health record dataset. Our findings revealed that the clinical setting, which included serum ammonia, had the highest predictive performance compared to the other feature sets. Furthermore, using the Bonferroni-adjusted method, the predictive accuracy of the feature set containing serum ammonia was significantly superior.

**Figure 4:**
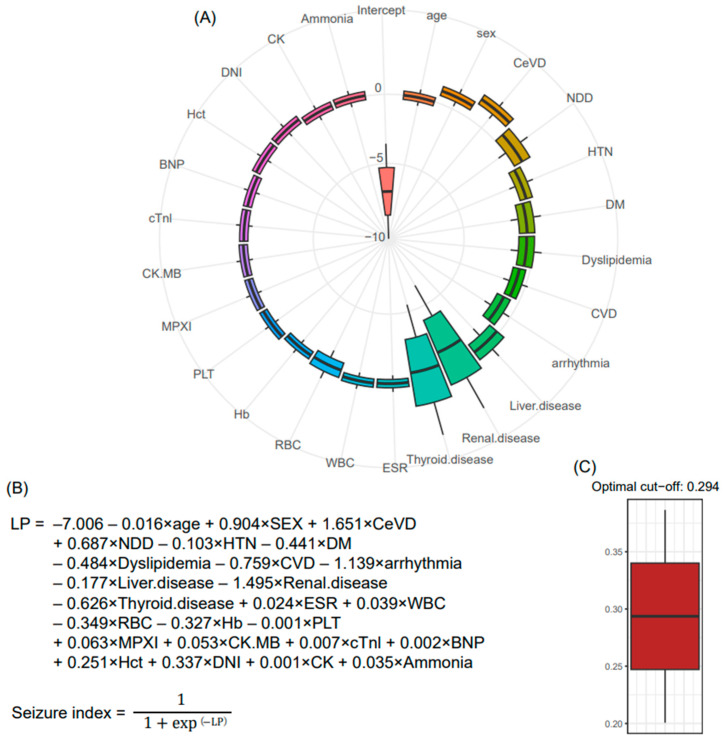
Final seizure classification model. (**A**) 1000 beta coefficients were determined for each biomarker using LR and a 250 × 4 CV approach. (**B**) The average value of the 1000 parameters was calculated for each predictor, forming the seizure classification model. The final model incorporated LR with both a linear unit and a non-linear unit (sigmoid function). (**C**) Through bootstrapping and F-score analysis, 1000 seizure index cut-offs were calculated. The mean value of these 1000 cut-offs (0.294) was determined as the optimal threshold for classifying seizure status from syncope; CeVD = cerebrovascular disease; NDD = neurodegenerative disease; HTN = hypertension; DM = diabetes mellitus; CVD = cardiovascular disease; ESR = erythrocyte sedimentation rate; WBC = white blood cell; RBC = red blood cell; Hb = hemoglobin; Plt = platelet; Hct = hematocrit; DNI = delta neutrophil index; MPXI = myeloperoxidase index; CK = creatinine kinase; CK.MB = creatinine kinase-myoglobin binding; cTnI = cardiac troponin I; BNP = brain-type natriuretic peptide; LR = logistic regression; CV = cross-validation.

The beta coefficients for each biomarker were determined using LR analysis. To determine the generalizable parameters, a 250 × 4 CV approach was employed, creating 1000 training sets. The LR was applied to these training sets, generating 1000 lists of coefficients that followed a Gaussian distribution, supporting the central limit theorem (Figure 4A). The average value from these distributions was determined as the final parameters for the seizure classification model (Figure 4B). The LR network consists of a linear unit and a non-linear unit (sigmoid function), producing output values between 0 and 1. To establish the robust cutoff value for the seizure index from the LR-based network, a bootstrapping method was implemented with 1000 iterations, resulting in 1000 distributions of F-scores. The corresponding maximum F-score values were extracted. Ultimately, the optimal cutoff value of 0.294 for the seizure index was determined (Figure 4C). By applying the final seizure classification model and the optimal cutoff value to the entire dataset, we evaluated the performance of the model using four indices: sensitivity, specificity, positive predictive value (PPV), and negative predictive value (NPV). Our model demonstrated favorable performance in terms of sensitivity, specificity, and PPV. However, it fell short in achieving satisfactory results for NPV (Table S2).

## 4. Discussion

The current study established a classification model to distinguish epileptic seizures, a major cause of transient loss of consciousness, from syncope through medical history-taking and routine blood tests conducted in the emergency room.

An expert review was conducted to select predictive factors to ensure the generalizability of the results, and statistical tests were used to determine the association between the 30 candidate factors and epileptic seizures. Among these, WBC, RBC, hemoglobin, hematocrit, DNI, creatinine kinase, and ammonia were significantly associated with epileptic seizures. However, when evaluated using our seizure classification model, most of these factors did not significantly improve the performance. Interestingly, serum ammonia level was the most relevant laboratory test for predicting epileptic seizures.

Epileptic seizures increase the body’s oxygen demand due to muscle contractions and catecholamine secretion. The increase in oxygen demand during seizures can lead to the release of lactate, ammonia, and CK from the damaged tissues and skeletal muscles. This process can trigger cytokine release and leukocytosis [18]. Previous studies have shown that muscle damage after GTCS can cause an elevation in CK levels. The elevation in CK levels has been used as a diagnostic tool to differentiate GTCS from syncope [19]. However, PNES associated with dynamic motor behavior was not associated with CK elevation [20]. The low sensitivity of CK as a diagnostic tool limits its use in clinical settings despite its usefulness [21].

Yanagawa et al. were the first to report an association between hyperammonemia and generalized convulsions [22]. Since then, transient hyperammonemia has emerged as a potential marker of post-ictal status and for predicting the presence of epileptic seizures [23]. A prospective study conducted in Korea, which included 65 seizure patients and 38 syncope patients, found that elevated plasma ammonia levels during acute post-ictal status could be useful in identifying seizures with a sensitivity of 0.65 and specificity of 0.80 [9]. Previous studies have reported that hyperammonemia lasts 3–8 h after a seizure and then returns to normal [24]. In this study, plasma ammonia samples collected within 8 h of hospital admission from patients who arrived at the emergency room with loss of consciousness were analyzed. Hyperammonemia may occur due to the overproduction or decreased metabolism of ammonia caused by medical conditions, such as liver disease. Therefore, a statistical machine-learning technique was used in this study to account for various medical conditions, including liver diseases.

When visiting the emergency room, a complete blood count (CBC) is one of the laboratory tests that can be quickly confirmed. In a study conducted in the United States, it was found that CBC was the most commonly performed blood test in patients with complaints of seizures and syncope, with rates of 66.4% and 64.8%, respectively [25]. It is well known that epileptic seizures cause a temporary increase in body temperature and inflammatory cytokines [18], which can lead to leukocytosis [26,27]. Consistent with our results, this is commonly induced in the post-ictal state [12]. However, among standard laboratory tests, the delta neutrophil index (DNI), which reflects the proportion of immature granulocytes in circulation, has not yet been studied as a possible marker of epileptic seizures. Recent studies have identified the DNI as a factor predicting the progression of sepsis in critically ill patients [28] and as a predictive factor for conditions other than cerebral infarction that can induce drowsiness [29]. Recent studies have shown that epileptic seizures increase the neutrophil-to-lymphocyte ratio [30,31]. In our study, although the automated model’s performance in predicting epileptic seizures was weak, we found a significant elevation in DNI in patients with epileptic seizures, suggesting an increase in the number of immature neutrophils in post-ictal conditions. However, further studies are required to confirm these findings.

Hematocrit measures the proportion of red blood cells in the blood and is included in the complete blood count. There are limited studies investigating the relationship between hemogram parameters and epileptic seizures [32]. Our study found that the epileptic seizure group showed a slight increase in RBC count and hematocrit in the post-ictal condition. However, elevated RBC and hematocrit levels did not show adequate predictive performance in the automated model used in our study. Further research is required to understand this relationship better.

Previous studies have shown that cardiac markers, including BNP, are not significantly elevated in the post-ictal state [8,33]. However, if it is elevated, comorbid cardiac problems should be considered [34]. In our study, the cardiac troponin I, BNP, and CK-MB levels were not significantly different between the epileptic seizure and syncope groups.

There are several laboratory tests that have been studied in the differential diagnosis of epileptic seizure that were not included in our study. One of these tests is serum lactate, formed by anaerobic glucose metabolism in the liver, and its elevation indicates tissue hypoxia. A study showed that patients with generalized tonic-clonic seizures exhibited a high elevation of serum lactate within two hours after the event compared to patients with syncope and psychogenic non-epileptic seizures [35]. However, serum lactate level was not considered in our study, as it is not frequently measured in patients visiting the emergency room. Another test studied for the differential diagnosis of epileptic seizures is prolactin. Epileptic seizures affect the hypothalamic–pituitary axis through connections between the limbic system and the hypothalamus. Therefore, prolactin has been used to differentiate epileptic seizures from psychogenic non-epileptic seizures since the 1970s [36]. However, prolactin was not included in our study because it has not been routinely studied in clinical practice. A recent study showed that syncope might also lead to prolactin elevation [37]. Serum NSE is a well-known biomarker used to estimate the prognosis of hypoxic brain injury after cardiac resuscitation [38]. Previous studies have shown that elevated NSE levels could be used to differentiate epileptic seizures from syncope [39]. However, our study did not measure serum NSE levels because it is not routinely performed as a basic blood test and is expensive.

This study has several advantages. One of the advantages of this study is that it confirmed the consistency of the diagnosis by matching the initial chief complaint with the final diagnosis made by a specialist. Referring to a previous study of emergency room patients, the percentage of cases in which the chief complaint and final diagnosis matched was only 51.7% for seizure patients and 68.5% for syncope patients [25]. Therefore, this study aimed to overcome this limitation by comparing diagnoses made by emergency medicine physicians with those made by neurologists and cardiologists during subsequent consultations. By ensuring the diagnostic precision through the involvement of specialists, it can be affirmed that the research results have achieved a heightened level of clarity. Secondly, this study exhibits a significant strength in its concurrent incorporation of diverse hematological tests capable of detecting previously identified post-seizure changes. Such a comprehensive examination of multiple variables holds practical value for future clinical applications, rendering it more realistic and applicable in a real-world setting. Finally, a machine-learning methodological approach with LR analysis was used to build a classification model. However, a recent study classified the model as a shallow classifier and argued that it could not effectively represent the complexity of actual clinical data [40]. One of the main reasons for this is the nonlinear relationship between independent and dependent variables, which LR has limitations in representing. We aimed to represent nonlinear data using random sampling techniques. Furthermore, this random data separation technique aimed to produce a more generalized performance by learning through a training dataset and calculating predictive performance using a validation dataset. Using this statistical method, our model showed that serum ammonia elevation had the strongest association with distinguishing epileptic seizures among patients with transient loss of consciousness in the emergency room.

This study has some limitations that need to be acknowledged. Firstly, this study lacks detailed clinical features for each episode of loss of consciousness. As a retrospective study based on records from emergency departments, it was difficult to consistently obtain specific descriptions beyond records indicating the presence of muscle contractions, including tonic-clonic components, for epileptic seizures. Considering previous studies suggesting that specific laboratory data may originate from muscle tissue damage, further research with accurate semiology analysis is needed to determine the significance of the results of this study in distinguishing nonconvulsive seizures from syncope. Secondly, a limitation of this study is the timing of blood sampling, which was within 8 h after the emergency department visit. It would have been ideal to verify the time elapsed from symptom onset to emergency department visit, but this information was not consistently documented in all emergency department records. Conducting a study that measures the total period from the onset of paroxysmal conditions to blood test taking would be more clinically applicable. Furthermore, potential cofounding factors such as medications taken by the patients that could influence the observed differences were not fully accounted for in the analysis. Lastly, the variable selection was only performed on the training dataset, and the statistical technique used in the intermediate stage of the variable selection process utilized all datasets, which may have affected the reliability of the results. Although previous studies have reported prediction models constructed without additional verification, it is still important to address the limitation of variable selection being performed only on the training dataset [41].

Despite these limitations, the significance of our study lies in the fact that it adds new evidence to previous research by utilizing a novel method, and our results are consistent with those of previous studies.

## 5. Conclusions

In our study, we observed that several laboratory blood tests, including WBC, RBC, hemoglobin, hematocrit, DNI, CK, and ammonia levels, were higher in patients with epileptic seizures than in those with syncope among patients with transient loss of consciousness who visited the emergency department. We developed a seizure classification model based on the results of our LR analysis and integrative selection of predictors. Our findings indicated that serum ammonia levels had the strongest association with epileptic seizures. Therefore, we recommend measuring ammonia levels in the emergency department as an effective approach for the early prediction of epilepsy.

## Figures and Tables

**Table 1:** Comparison of clinical and laboratory variables between patients with syncope and seizure.

	Syncope (*n* = 170)	Seizure (*n* = 90)	*p*-Value
Age, years	59.5 ± 1.54	55.4 ± 1.76	0.105
Male	79 (46.5)	54 (60)	0.052
Cerebrovascular disease	23 (13.5)	31 (34.4)	<0.001
Neurodegenerative disease	10 (5.9)	6 (6.7)	1
Hypertension	43 (25.3)	21 (23.3)	0.843
Diabetes mellitus	26 (15.3)	10 (11.1)	0.459
Dyslipidemia	30 (17.6)	8 (8.9)	0.086
Cardiovascular disease	30 (17.6)	12 (13.3)	0.47
Cardiac arrhythmia	24 (14.1)	8 (8.9)	0.307
Liver disease	10 (5.9)	10 (11.1)	0.207
Renal disease	14 (8.2)	3 (3.3)	0.209
Thyroid disease	10 (5.9)	3 (3.3)	0.55
Erythrocyte sedimentation rate (mm/h)	9 ± 0.8	10.6 ± 1.21	0.255
White blood cell counts (×10^3^/μL)	7.6 ± 0.23	9.9 ± 0.48	<0.001
Red blood cell counts, (×10^6^/μL)	4.4 ± 0.04	4.5 ± 0.07	0.029
Hemoglobin (g/dL)	13.4 ± 0.15	14 ± 0.19	0.013
Hematocrit (%)	40 ± 0.4	42.4 ± 0.62	0.002
Platelet counts (×10^3^/μL)	230.3 ± 5.22	235.6 ± 10.49	0.647
Delta neutrophil index (%)	0.3 ± 0.06	0.7 ± 0.18	0.017
Myeloperoxidase index	0.9 ± 0.32	1.1 ± 0.47	0.8
Creatinine kinase (IU/L)	132 ± 12.75	336.1 ± 79.45	0.013
Creatinine kinase-myoglobin binding (ng/mL)	1.6 ± 0.13	4.6 ± 1.7	0.084
Cardiac troponin I (ng/mL)	20.2 ± 13.59	63 ± 34.58	0.252
Brain-type natriuretic peptide (pg/mL)	63 ± 9.66	95.7 ± 20.85	0.158
Ammonia (µg/dL)	29.2 ± 1.45	93.9 ± 12.79	<0.001

Continuous and categorical variables are described as mean ± SD and number (percent), respectively; SD = standard deviation.

## Data Availability

The raw data supporting the conclusions of this article will be made available by the authors, without undue reservation.

## Supplementary Materials

The following supporting information can be downloaded at: https://www.mdpi.com/article/10.3390/jcm12124031/s1, Table S1: References in the literature-based search for variables; Table S2: Sensitivity, specificity, positive predictive value, and negative predictive value for Feature 3, Feature 4, and Feature 5 (final seizure classification model).
