# Supplementary material for: Classification Model for Epileptic Seizure Using Simple Postictal Laboratory Indices
Source: J Clin Med. 2023 Jun 13;12(12):4031. doi: 10.3390/jcm12124031 (PMC10298879; doi:10.3390/jcm12124031)
Supplement: Supplementary file 1 [file jcm-12-04031-s001.zip › jcm-2403061-supplementary.pdf]

*Supplementary Material*

# Classification Model for Epileptic Seizure Using Simple Postictal Laboratory Indices

**Sun Jin Jin [1], Taesic Lee [2,3], Hyun Eui Moon [3], Eun Seok Park [1], Sue Hyun Lee [1], Young Il Roh [4], Dong Min Seo [5], Won-Joo Kim [6] and Heewon Hwang [1,*]**

[1] Department of Neurology, Wonju Severance Christian Hospital, Yonsei University Wonju College of Medicine, Wonju 26426, Republic of Korea
[2] Division of Data Mining and Computational Biology, Institute of Global Health Care and Development, Wonju 26426, Republic of Korea
[3] Department of Family Medicine, Yonsei University Wonju College of Medicine, Wonju 26426, Republic of Korea
[4] Department of Emergency Medicine, Yonsei University Wonju College of Medicine, Wonju 26426, Republic of Korea
[5] Department of Medical Information, Yonsei University Wonju College of Medicine, Wonju 26426, Republic of Korea
[6] Department of Neurology, Gangnam Severance Christian Hospital, Yonsei University College of Medicine, Seoul 06273, Republic of Korea
* Correspondence: nmdrhee@yonsei.ac.kr; Tel.: +82-33-741-2376; Fax: +82-33-741-0520

**Table S1.** References in the literature-based search for variables.

| Variables | Journal | Author | Year of publication |
|---|---|---|---|
| Age | Epilepsy in older people | Sen et al. | 2020 |
| Sex | Sex differences in seizure types and symptoms | Carison et al. | 2014 |
| Cerebrovascular disease | Epilepsy in cerebrovascular diseases: Review of experimental and clinical data with meta-analysis of risk factors | Ferlazzo et al. | 2016 |
| Neurodegenerative disease | Dementia and adult-onset unprovoked seizures | Hesdorffer et al. | 1996 |
| Hypertension | Hypertension and the risk of new-onset unprovoked seizures. | Ng et al. | 1993 |
| Diabetes mellitus | Association Between Seizures and Diabetes Mellitus: A Comprehensive Review of Literature | Yun et al. | 2013 |
| Dyslipidemia | A high risk of hyperlipidemia in epilepsy patients: a nationwide population-based cohort study | Harnod et al. | 2014 |
| Cardiovascular disease | Association Between Midlife Risk Factors and Late-Onset Epilepsy Results From the Atherosclerosis Risk in Communities Study | Johnson et al. | 2018 |
| Cardiac arrhythmia | Cardiac arrhythmias during or after epileptic seizures | van der Lende et al. | 2016 |
| Liver disease | Risk of seizures and status epilepticus in older patients with liver diseases | Alkhachroum et al. | 2018 |
| Renal disease | Subclinical seizure activity and prophylactic phenytoin infusion in acute liver failure: a controlled clinical. | Ellis et al. | 2000 |
| Thyroid disease | Thyroid hormones: Possible roles in epilepsy pathology | Tamijani et al. | 2015 |
| Erythrocyte sedimentation rate | Predictors of early seizure recurrence in patients admitted for seizures in the emergency department | Choquet et al. | 2008 |
| White blood cell | Peripheral WBC count and serum prolactin level in various seizure types and nonepileptic events | Shah et al. | 2001 |
| Red blood cell | A study on the relationship between CBC and EEG for epilepsy patients | Jo et al. | 2015 |
| Hemoglobin | A study on the relationship between CBC and EEG for epilepsy patients | Jo et al. | 2015 |
| Hematocrit | A study on the relationship between CBC and EEG for epilepsy patients | Jo et al. | 2015 |
| Platelet | A study on the relationship between CBC and EEG for epilepsy patients | Jo et al. | 2015 |
| Delta neutrophil index | Delta neutrophil index: in search of an early indicator of sepsis | Peneva et al. | 2021 |
| Myeloperoxidase index | Evaluation of usefulness of myeloperoxidase index (MPXI) for differential diagnosis of systemic inflammatory response syndrome (SIRS) in the emergency department | Cha et al. | 2014 |
| Creatinine kinase | Serum creatine phosphokinase is helpful in distinguishing generalized tonic-clonic seizures from psychogenic nonepileptic seizures and vasovagal syncope | Petramfar et al. | 2009 |
| Creatinine kinase-myoglobin binding | Elevated CK-MB mass and plasma brain-type natriuretic peptide concentrations following convulsive seizures in children and adolescents: Possible evidence of subtle cardiac dysfunction | Alehan et al. | 2009 |
| Cardiac troponin I | Cardiac involvement in patients with acute neurologic disease: confirmation with cardiac troponin I. | Dixit et al. | 2000 |
| Brain-type natriuretic peptide | cTnI, BNP and CRP profiling after seizures in patients with drug-resistant epilepsy | Faria et al. | 2020 |
| Ammonia | Postictal transient hyperammonemia as an indicator of seizure disorder. | Liu et al. | 2010 |
| Prolactin | Predictivity of Plasma Prolactin Levels in Differentiating Epilepsy from Pseudoseizures: A Prospective Study | Anzola et al. | 1993 |
| Lactate | Lactate as a diagnostic marker in transient loss of consciousness | Matz et al. | 2016 |
| Myoglobin | Impairment of renal function after generalised seizures | Nielsen et al. | 1987 |
| Neuron specific enolase | Serum neuron-specific enolase level as a biomarker in differential diagnosis of seizure and syncope | Lee et al. | 2010 |
| Protein S100-B | Clinical significance of serological biomarkers and neuropsychological performances in patients with temporal lobe epilepsy | Chang et al. | 2012 |

**Table S2.** Sensitivity, specificity, positive predictive value, and negative predictive value for Feature 3, Feature 4, and Feature 5 (final seizure classification model).

| | Sensitivity | Specificity | Positive predictive value | Negative predictive value |
|---|---|---|---|---|
| **Feature 3** | 0.700 | 0.733 | 0.832 | 0.564 |
| **Feature 4** | 0.729 | 0.756 | 0.849 | 0.596 |
| **Feature 5 (Final seizure classification model)** | 0.771 | 0.811 | 0.885 | 0.652 |
